# Stingless bee honey alleviates cognitive deficits and hippocampal neurodegeneration in an Alzheimer's model: Behavioural, neurochemical, and histological analyses

**DOI:** 10.3934/Neuroscience.2026001

**Published:** 2026-01-16

**Authors:** Shah Rezlan Shajahan, Zaw Myo Hein, Hussin Muhammad, Mohd Zulkifli Mustafa, Yatinesh Kumari, Imrana Jazuli, Azlina Zulkapli, Norshafarina Shari, Che Mohd Nasril Che Mohd Nassir, Muhammad Danial Che Ramli

**Affiliations:** 1 School of Graduate Studies, Postgraduate Centre, Management and Science University, Shah Alam 40100, Selangor, Malaysia; 2 Department of Basic Medical Sciences, College of Medicine, Ajman University, P.O.BOX:346 Ajman, United Arab Emirates; 3 Toxicology & Pharmacology Unit, Herbal Medicine Research Centre, Institute for Medical Research, National Institutes of Health, Shah Alam 40170, Selangor, Malaysia; 4 Department of Neuroscience, School of Medical Sciences, Universiti Sains Malaysia, Kubang Kerian, 16150 Kota Bharu, Kelantan, Malaysia; 5 Neurological Disorder & Aging Research Group (NDA), Neuroscience Research Strength (NRS), Jeffrey Cheah School of Medicine and Health Sciences, Monash University Malaysia, 47500 Selangor, Malaysia; 6 Laboratory Animal Resource Unit, Special Resource Centre, Institute for Medical Research, National Institute of Health, Jalan Pahang, 50588 Kuala Lumpur, Malaysia; 7 Department of Anatomy and Physiology, School of Basic Medical Sciences, Faculty of Medicine, University Sultan Zainal Abidin, 20400 Kuala Terengganu, Terengganu, Malaysia; 8 Faculty of Health & Life Sciences, Management & Science University, Shah Alam 40100, Selangor, Malaysia

**Keywords:** Alzheimer's disease, stingless bee honey, cognitive impairment, aluminium chloride, D-galactose, hippocampus, acetylcholinesterase, dopamine, serotonin, corticosterone

## Abstract

Stingless bee honey (SBH), widely consumed in Southeast Asia, is traditionally valued for its medicinal and nutritional properties, particularly in promoting brain health. However, its neuroprotective potential against Alzheimer's disease (AD) remains underexplored. In this study, we investigated the therapeutic effects and safety of SBH in a rat model of AD. A total of sixty-three adult male Sprague-Dawley rats (180–200 g) were used: Fifteen were assigned to three toxicity groups (500, 750, 1000 mg/kg; *n* = 5) and forty-eight to six therapeutic groups (*n* = 8): Normal control, AD (AlCl₃ + D-gal), AD + Donepezil (1.5 mg/kg), and three SBH-treated groups (500, 750, 1000 mg/kg). Alzheimer-like pathology was induced by aluminium chloride (150 mg/kg) and D-galactose (300 mg/kg), followed by 14 days of treatment. Toxicity was evaluated through liver and kidney histopathology, while behavioural performance was assessed using the Open Field Test and Morris Water Maze. Serum dopamine, serotonin, corticosterone, and acetylcholinesterase activity were quantified via ELISA, and hippocampal morphology was examined histologically. SBH administration produced no signs of systemic toxicity and significantly improved exploratory activity and spatial learning, with the most pronounced effects at 750 mg/kg. Biochemical assays showed reduced acetylcholinesterase and corticosterone levels alongside increased dopamine and serotonin concentrations. Histological analysis confirmed neuronal preservation and reduced hippocampal damage. Inclusion of Donepezil as a positive control enabled comparison with a standard pharmacological treatment. These findings demonstrated that SBH is a safe and promising natural therapeutic capable of alleviating cognitive deficits associated with AD.

## 1. Introduction

Alzheimer's disease (AD) is a progressive neurological disorder and a major global public health challenge, primarily affecting memory, cognition, and behaviour. As the most prevalent form of dementia, AD accounts for up to 70% of all cases and is pathologically defined by the accumulation of amyloid-beta (Aβ) plaques and neurofibrillary tangles composed of tau protein in the brain. The disease disproportionately affects the elderly population, with approximately one in eight individuals over the age of 65 at risk of developing the condition [1],[2].

Epidemiological projections suggest that the number of people living with AD will triple by 2050, creating a significant burden on healthcare systems and families worldwide [3],[4]. The clinical course of AD is typically gradual, progressing from mild memory disturbances to severe cognitive impairment and personality changes. Early symptoms may include difficulties in language, daily functioning, and recent memory retention, while more advanced stages are often marked by agitation, loss of independence, and impaired mobility [3],[4].

As of 2024, over 55 million individuals globally are living with dementia, with nearly 10 million new cases reported annually. AD remains the leading cause, accounting for 60%–70% of all dementia cases. The global dementia population is expected to rise to 78 million by 2030 and 139 million by 2050, with the sharpest increase predicted in low- and middle-income countries. Currently, 60% of dementia patients reside in these regions a figure projected to grow to 71% by mid-century [5] .

The economic impact is equally alarming. Dementia-related costs are estimated to exceed USD 1.3 trillion annually and are expected to surge to USD 2.8 trillion by 2030. A significant portion of this expenditure stems from informal care, often provided by family members, which accounts for roughly 40% of total costs worldwide [5].

Despite extensive research, pharmacological treatments for AD such as cholinesterase inhibitors and the NMDA receptor antagonist memantine offer only symptomatic relief and do not prevent or reverse disease progression. Consequently, interest has shifted towards the exploration of natural products as safer, multi-targeted alternatives [6]. Several plant-derived compounds have demonstrated promising therapeutic effects. For example, Aloe vera has been explored for its neuroactive phytochemicals, while berberine, an alkaloid found in *Argemone mexicana* and *Coptis chinensis*, exhibits anti-inflammatory, antioxidant, and cognitive-enhancing properties [6],[7].

Honey has been used for centuries in various traditional medicine systems across Asia for its healing and health-promoting properties. Stingless bee honey (SBH), derived from *Heterotrigona itama*, holds a significant place in Malay, Thai, and Indonesian ethnomedicine. Traditionally, it is consumed to improve vitality, treat gastrointestinal ailments, enhance wound healing, and support cognitive health in aging populations [8]. Among indigenous Malaysian communities, (SBH) is considered a “functional tonic” believed to promote memory and brain function. Its use as a folk remedy reflects both its nutritional and medicinal value, particularly in the context of age-related cognitive decline, thereby justifying its evaluation in modern scientific models of neurodegeneration such as AD.

Among natural products, SBH has emerged as a compound of particular interest due to its rich composition of flavonoids, catechins, and other bioactive compounds. These constituents are known to exert antioxidant, anti-inflammatory, and neurofunctional effects, potentially mitigating oxidative stress, inflammation, and cognitive decline associated with age-related neurodegenerative diseases, including AD [3],[8]. Studies have shown that honey can modulate biological pathways relevant to AD pathogenesis, such as gene expression related to neuroinflammation and amyloid genesis [4],[9],[10]. However, most of these investigations have focused on conventional honey types, leaving a gap in the understanding of SBH's unique effects in neurodegenerative contexts.

(SBH) is rich in diverse bioactive constituents, including polyphenols, flavonoids, phenolic acids, and organic acids, all recognised for their antioxidant and neuroactive properties [11],[12]. Among these, flavonoids such as quercetin, luteolin, and apigenin play crucial roles in scavenging free radicals, enhancing synaptic plasticity, and modulating cholinergic neurotransmission by inhibiting acetylcholinesterase activity mechanisms that are disrupted in AD. Likewise, phenolic acids such as caffeic and gallic acids exert neuroprotective effects by attenuating neuroinflammation and preserving mitochondrial function. These bioactive compounds collectively contribute to oxidative stress reduction, neurotransmitter regulation, and enzyme inhibition, which may underlie the cognitive and biochemical improvements observed in experimental models.

To address the limited understanding of SBH's therapeutic potential in AD, we investigate its effects in a rat model of AD induced by aluminium chloride (AlCl₃) and D-galactose (D-gal), two agents known to induce oxidative stress, neuroinflammation, and cognitive dysfunction. To provide a standard pharmacological reference, Donepezil, a widely used acetylcholinesterase inhibitor, was included as a positive control. Clinically, Donepezil is approved for the symptomatic management of memory loss and cognitive decline in AD by enhancing cholinergic neurotransmission in the brain [11],[13],[14]. Including Donepezil enables a direct comparison of the therapeutic efficacy of SBH against a recognised standard treatment, thereby contextualizing the potential of SBH as a natural neuroprotective agent. We aim to provide a comprehensive evaluation of SBH as a natural, multi-targeted therapeutic agent for mitigating cognitive impairment and neurodegeneration in AD.

## 2. Materials and methods

### 2.1. Source of honey

Multiflora SBH used in this study was obtained from a reputable stingless bee farm at University Sains Malaysia (USM), Health Campus, Kubang Kerian, Kelantan (6.0560° N, 102.2905° E). The honey was produced by *Heterotrigona itama* (Hymenoptera: Apidae: Meliponini), harvested using standard contamination-free procedures [8]. Each batch was filtered and stored at 4°C until use. Quality control analyses were performed to ensure uniformity in pH, moisture content, and physicochemical parameters prior to administration [15].

### 2.2. Justification of the AD animal model

The Alzheimer's-like condition was induced in rats via co-administration of aluminium chloride (AlCl₃; 150 mg/kg) and D-galactose (D-gal; 300 mg/kg) once daily for seven consecutive days, according to established protocols [16],[17]. AlCl₃ and D-gal were dissolved in 0.9% saline and administered orally. This dosage combination has been widely used to produce consistent cognitive and biochemical impairments in rodent AD models.

### 2.3. Population and sample

A total of sixty-three adult male Sprague-Dawley rats (aged 8 weeks; 180–200 g) were used. Animals were procured from the Institute for Medical Research (IMR), Malaysia, and acclimatised for one week under standardised conditions (22 ± 2°C, 12-hour light/dark cycle) with ad libitum access to chow and water. Acclimatization minimised transportation-induced stress and physiological variability prior to experimental procedures [18].

### 2.4. Experimental groups

The rats were randomly assigned to nine experimental groups, each designed to test various aspects of the study hypothesis. This random allocation was crucial in preventing selection bias and ensuring comparability between the different treatment arms at the outset of the experiment. The formation of groups and their respective treatment assignments have been summarised in Tables 1 and 2.

As in Table 1, to evaluate the short-term toxicity profile of SBH, fifteen healthy rats were divided into three groups (*n* = 5 per group). These rats were not subjected to AD induction. These groups were monitored for behavioural signs of toxicity and subjected to liver and kidney histopathological evaluations post-treatment. The doses of 500, 750, and 1000 mg/kg of SBH were selected based on previous studies demonstrating its safety in rodents. According to the OECD Test Guideline 425 (2022) [19], substances that produce no adverse effects at an oral dose of 2000 mg/kg are considered non-toxic. Consistent with this classification, researchers have reported no signs of toxicity or mortality in rodents administered SBH at comparable doses, further supporting its safe use in experimental models [20],[21].

**Table 1.** Subacute toxicity assessment groups of stingless bee honey (SBH) in rats.

**Group**	***n***	**Treatment**	**Experimental design**
1	5	Subacute Toxicity	SBH 500 mg/kg–Low dose
2	5	Subacute Toxicity	SBH 750 mg/kg–Intermediate dose
3	5	Subacute Toxicity	SBH 1000 mg/kg–High dose

Notes: *n*: Sample size, SBH: Stingless Bee Honey, Toxicity was assessed *in vivo* through histopathological evaluation of liver and kidney tissues, in accordance with OECD Test Guideline.

**Table 2.** Drug dose, treatment doses and administration of groups.

**Group**	***n***	**Treatment**	**Experimental design**
1	8	Control Normal	Saline
2	8	Negative (AD)	AlCl₃ (150 mg/kg) + D-gal (300 mg/kg)
3	8	Positive (AD + Donepezil)	AlCl₃ (150 mg/kg) + D-gal (300 mg/kg) + Donepezil (1.5 mg/kg)
4	8	Treatment 1	AlCl₃ (150 mg/kg) + D-gal (300 mg/kg) + SBH (500mg/kg)
5	8	Treatment 2	AlCl₃ (150 mg/kg) + D-gal (300 mg/kg) + SBH (750 mg/kg)
6	8	Treatment 3	AlCl₃ (150 mg/kg) + D-gal (300 mg/kg) + SBH (1000 mg/kg)

Notes: AD: Alzheimer's Disease, AlCl₃: Aluminium Chloride, D-gal: D-galactose, *n*: Sample size, SBH: Stingless Bee Honey.

As shown in Table 2, forty-eight rats were randomly assigned into six groups (*n* = 8 per group) for AD induction and subsequent therapeutic assessment. Aluminium chloride (AlCl₃; 150 mg/kg) and D-galactose (D-gal; 300 mg/kg) were used to induce cognitive impairment over 7 days. Treatment regimens were administered for 14 days post-induction. AlCl₃ and D-gal were prepared in 0.9% saline, while Donepezil was dissolved in distilled water. SBH was administered orally at the respective dosages. Following the treatment period, all groups underwent behavioural testing (Open Field Test and Morris Water Maze), followed by ELISA analyses and Histology analysis.

Additionally, the toxicity and therapeutic study groups consisted of distinct animal cohorts. The fifteen rats in the toxicity study were healthy and used exclusively to assess the safety of SBH at various doses. These animals were not subjected to AD induction. In contrast, the remaining 48 rats were used to establish the AD model and evaluate the therapeutic efficacy of SBH and Donepezil. This separation ensured clear differentiation between the toxicological safety profile and the therapeutic potential of SBH, thereby enhancing the ethical integrity and scientific validity of the experimental design.

### 2.5. Subacute in vivo toxicity evaluation

At the end of the SBH dosing regimen, rats were humanely euthanised, and liver and kidney tissues were collected for histopathological examination to assess potential subacute toxicity at the organ level. Tissues were fixed in 10% buffered formalin, dehydrated through graded alcohols, cleared with xylene, and embedded in paraffin. Sections were cut at 5 µm thickness using a microtome and stained with haematoxylin and eosin (H&E) following standard protocols [22],[23]. Microscopic analysis focused on detecting structural abnormalities such as hepatocellular degeneration, sinusoidal dilation, tubular necrosis, glomerular shrinkage, and inflammatory infiltration. Across all groups, liver and kidney sections displayed preserved architecture with no significant pathological changes, indicating that SBH did not induce histological toxicity at the tested doses.

### 2.6. Biochemical and behavioural validation of the Alzheimer's disease model

To ensure the successful establishment of an AD model in rats, a comprehensive validation was performed using behavioural and biochemical assessments. The AD-like condition was induced through the combined administration of aluminium chloride (AlCl₃) and D-galactose (D-gal), agents known to synergistically promote oxidative stress, neuroinflammation, and cognitive deficits resembling those observed in human AD pathology. Behavioural assessments were conducted using the Open Field Test (OFT) and the Morris Water Maze (MWM). These tests were used to evaluate anxiety-like behaviours, locomotor activity, spatial learning, and memory retention. In the OFT, the AD-induced rats exhibited a marked reduction in exploratory behaviours, including fewer line crossings and rearing episodes, indicating increased anxiety and reduced spontaneous activity. In the MWM, the same group demonstrated significantly prolonged escape latency during the acquisition trials and reduced time spent in the target quadrant during the probe trial, suggesting impairments in learning and memory functions [20].

To complement behavioural validation, biochemical analysis was conducted using enzyme-linked immunosorbent assay (ELISA) to measure the levels of four key neurochemical markers: dopamine, corticosterone, acetylcholinesterase (AChE), and serotonin. The results revealed a significant decrease in dopamine levels among the AD-induced rats, which reflects impaired dopaminergic signalling commonly associated with cognitive dysfunction and motivational decline. Corticosterone levels were markedly elevated, indicating activation of the hypothalamic pituitary adrenal (HPA) axis in response to chronic stress, which is known to exacerbate neuronal damage and memory loss. Furthermore, AChE activity was substantially increased in the AD group, highlighting the disruption of cholinergic neurotransmission a core feature of Alzheimer's pathology. Last, serotonin levels were observed to be significantly reduced, which may contribute to mood-related disturbances and cognitive deficits in the disease model. Together, the behavioural and biochemical findings confirm the successful induction of an Alzheimer's-like phenotype in the experimental animals. These outcomes establish a reliable platform to assess the therapeutic efficacy of SBH in alleviating cognitive dysfunction associated with AD [21].

In addition to the behavioural and biochemical findings, structural alterations in the hippocampus were observed through H&E staining, offering further support for successful AD model induction. The AlCl₃ and D-galactose treated group exhibited signs of neuronal damage, including reduced neuronal density, disrupted cellular arrangement, and the presence of pyknotic nuclei. These morphological changes are consistent with neurodegenerative processes and contribute to the anatomical validation of the Alzheimer's-like condition induced in this study.

### 2.7. Behavioural assessment

Cognitive and anxiety-like behaviours were assessed using the Open Field Test (OFT) and MWM. Prior to the behavioural assessments, all rats were acclimatised to the testing room for at least one hour to minimise environmental stressors. All behavioural procedures were conducted between 11:00 a.m. to 3:00 p.m. to ensure consistency and reduce variability due to circadian influences [24]–[26].

#### 2.7.1. Open field test (OFT)

The test was performed using a square wooden arena measuring 80 × 80 × 40 cm. The floor of the arena was marked with black lines to divide it into 16 equal squares, each measuring 4 × 4 cm. The walls were painted red, and the floor was white and polished to minimise distraction and enhance contrast [18],[26].

Each rat was placed gently at the centre of the arena and observed for three minutes. The behavioural parameters recorded included the number of lines crossed (to measure horizontal activity), frequency of rearing (standing on hind limbs, indicating exploratory behaviour), grooming episodes (self-cleaning behaviour linked to emotional status), and the number of faecal pellets (an indicator of stress or anxiety). This test provided insight into both motor coordination and anxiety-like symptoms. Reduced locomotor activity and exploratory behaviour such as fewer line crossings or less rearing were interpreted as indicative of neurodegeneration and anxiety, typical in AD-like states. Conversely, increased exploration and reduced emotional stress responses following SBH treatment suggested a reversal of AD-like symptoms and improvement in overall behavioural function [24],[27].

#### 2.7.2. Morris water maze

Spatial learning and memory retention were evaluated in a large circular pool measuring 1.5 m in diameter and 45 cm deep, filled with water rendered opaque using a non-toxic black dye to conceal a submerged escape platform. The hidden platform, measuring 10 × 10 cm, was placed 2 cm below the water surface in the southwest quadrant of the tank. Visual cues such as distinctive shapes and objects were placed around the testing chamber to serve as navigational aids for spatial learning. The testing environment was uniformly lit, and a ceiling-mounted video camera recorded all trials for later analysis [27]–[29].

The MWM was conducted in two phases: acquisition and probe. During the acquisition phase, each rat underwent four trials per day for four consecutive days. In each trial, the rat was released from one of four starting points at the edge of the pool and given 60 seconds to locate the hidden platform. If the platform was found, the rat was allowed to remain there for 15 seconds before removal. If the rat failed to find the platform within the allotted time, it was gently guided to it and allowed to stay for the same duration. This phase measured the learning ability of the rats across repeated trials.

Twenty-four hours after the final acquisition session, the probe test was conducted. In this phase, the platform was removed, and the rats were allowed to swim freely for 60 seconds. The amount of time spent in the target quadrant where the platform had previously been located was used as an index of memory retention. Higher time spent in the target zone indicated better memory performance, while lower values were associated with cognitive impairment [27]. The MWM test thus provided robust quantitative data on the cognitive capabilities of rats under various treatment conditions. Improvement in escape latency, time spent in the target quadrant, and path efficiency among SBH-treated rats was indicative of the honey's therapeutic potential in ameliorating AD-related cognitive deficits.

### 2.8. ELISA

To investigate the biochemical alterations associated with AD and to evaluate the potential therapeutic impact of multiflora SBH, ELISA was employed as a sensitive and specific method for quantifying key neurochemical biomarkers. The selected biomarkers dopamine, corticosterone, acetylcholinesterase (AChE), and serotonin play critical roles in the pathogenesis and progression of AD. Commercially available ELISA kits were used to ensure standardised and reproducible results, and all procedures were carried out according to the manufacturer's protocols, as outlined by Alhajj [30]. These biomarkers were measured from serum samples obtained from the rats at the end of the experimental period.

A total of 50 µL of each serum sample, along with standards and quality controls, was pipetted into wells pre-coated with antibodies specific to the target analyte. This was followed by the addition of a secondary detection antibody, forming a sandwich complex. The plates were then incubated at 37°C for 1 hour to allow proper binding and reaction. Following incubation, the wells were washed 3 to 5 times to remove any unbound substances and to reduce background noise. Subsequently, 100 µL of substrate solution was added to initiate the enzymatic reaction. The plates were incubated for an additional 30 minutes at room temperature in the dark, allowing the enzyme-substrate reaction to produce a detectable colour change. Finally, the reaction was stopped by adding 50 µL of stop solution to each well. The colour intensity, which is directly proportional to the concentration of the target analyte in the sample, was measured using a microplate reader set at 450 nm [30].

#### 2.8.1. Mechanistic implications

Changes in neurotransmitters and AChE activity were interpreted to reflect SBH's potential neuroprotective mechanisms. Elevation of dopamine and serotonin, reduction of corticosterone, and decreased AChE activity suggest modulation of cholinergic transmission, stress hormone regulation, and neurotransmitter balance. These effects are consistent with SBH's reported antioxidant and anti-inflammatory properties [28].

### 2.9. Histological assessment of the hippocampus

Following behavioural and biochemical analyses, histological evaluation of the hippocampal region was conducted to assess morphological changes associated with Alzheimer-like pathology. At the end of the treatment period, rats were anesthetised and sacrificed via transcranial perfusion using phosphate-buffered saline (PBS), followed by 10% neutral buffered formalin. Brains were carefully removed and post-fixed in formalin for 24 to 48 hours. Subsequently, tissues were dehydrated through a graded ethanol series, cleared in xylene, and embedded in paraffin. Coronal sections of the hippocampus (5 µm thick) were obtained using a rotary microtome. The sections were mounted on glass slides, deparaffinised, and rehydrated. Standard H&E staining was performed to visualise general cellular morphology. Stained sections were examined under a light microscope to evaluate neuronal integrity, cell density, and the presence of histopathological features. Images were captured using a digital imaging system for documentation and analysis. This histological procedure enables qualitative assessment of neurodegenerative changes and has been widely applied in experimental models of AD to validate neuronal damage and treatment efficacy [23],[27],[31].

### 2.10. Statistical analysis

Data analysis was conducted using GraphPad Prism version 9, Microsoft Excel, and Smart Software to ensure precision and reliability in evaluating the effects of SBH in an AD rat model. Results were expressed as mean ± standard error of the mean (SEM), with statistical significance set at *p* < 0.05 [11]. One-way ANOVA was used to compare group differences across behavioural and biochemical outcomes, followed by Tukey's post hoc test to identify specific intergroup variations. These analyses enabled the assessment of the dose-dependent impact of SBH on cognitive function and neurochemical markers. Descriptive statistics supported the identification of trends and outliers across treatment groups. For biochemical evaluation, ELISA absorbance values at 450 nm were converted to concentrations using standard curves. Comparisons of dopamine, corticosterone, acetylcholinesterase (AChE), and serotonin levels revealed that SBH treatment significantly modulated stress and neurotransmitter profiles, aligning with observed behavioural improvements. This rigorous statistical approach supported our conclusions, reinforcing SBH's therapeutic potential in mitigating cognitive deficits and neurochemical imbalances associated with AD [11].

### 2.11. Ethics approval of research

This study was conducted in accordance with institutional and international ethical guidelines for animal research. All experimental procedures involving animals were reviewed and approved by the Management and Science University Research Ethics Committee (MSU-REC), Malaysia, under approval number MSU–RMC–021–FR01–08–C3/017. The committee approved all aspects of the study, including the use of animals, handling procedures, treatment administration, and behavioural testing protocols.

## 3. Results

### 3.1. Renal histopathological assessment of subacute SBH toxicity in SD rats

**Figure 1.**
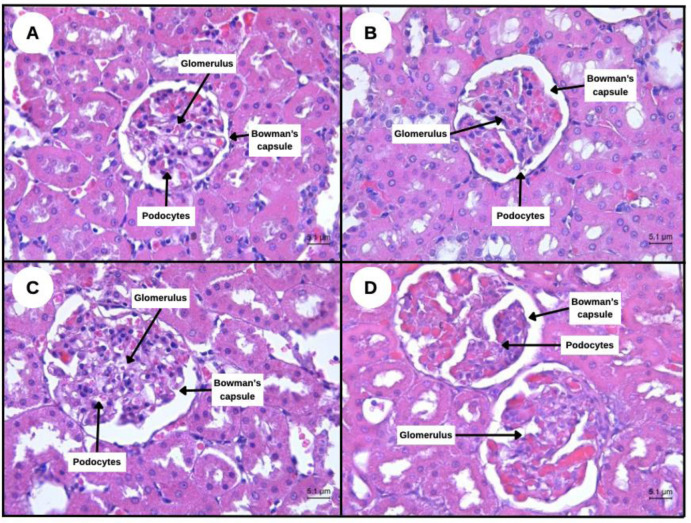
Representative photographs of rat kidney by light microscope with H&E staining at 40× magnification. (A) control rat kidney, (B) SBH (500 mg/kg) orally treated rat kidney showing the normal appearance, (C) SBH (750 mg/kg) orally treated rat kidney showing the normal appearance morphology, and (D) SBH (1000 mg/kg) orally treated rat kidney showing the normal appearance.

As shown in Figure 1, histopathological evaluation using H&E staining revealed no signs of subacute renal toxicity across all treatment groups receiving SBH. Kidney tissues were examined under light microscopy (40× magnification) for structural abnormalities, including glomerular or tubular damage, necrosis, inflammation, or fibrosis.

- **Control Group:** Showed normal renal architecture with intact glomeruli and defined tubules. No pathological alterations were observed.
- **SBH 500 mg/kg:** Histological features were comparable to controls. Glomeruli and tubules retained normal morphology with no signs of necrosis, degeneration, oedema, or inflammatory infiltration.
- **SBH 750 mg/kg:** Displayed preserved glomerular and tubular architecture, with no epithelial damage or interstitial pathology.
- **SBH 1000 mg/kg:** Even at the highest dose, renal tissue showed intact structural integrity with no evidence of glomerulosclerosis, tubular necrosis, or fibrosis.

These findings confirm that subacute administration of SBH up to 1000 mg/kg does not induce nephrotoxicity, supporting its renal safety for therapeutic applications.

### 3.2. Liver histopathological assessment of subacute SBH toxicity in SD rats

**Figure 2.**
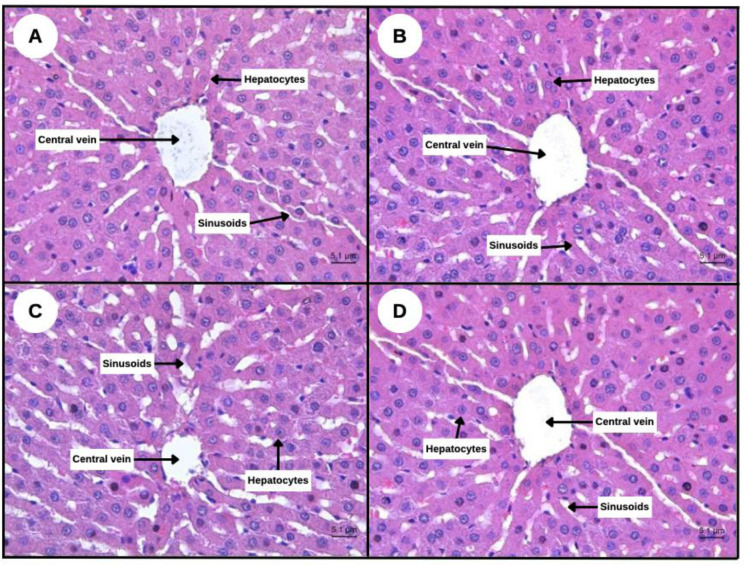
Representative photographs of rat liver by light microscope with H&E staining at 40× magnification. (A) control rat liver, (B) SBH (500 mg/kg) orally treated rat liver showing the normal appearance, (C) SBH (750 mg/kg) orally treated rat liver showing the normal appearance morphology, and (D) SBH (1000 mg/kg) orally treated rat liver showing the normal appearance.

As shown in Figure 2, Histopathological analysis using H&E staining was conducted to evaluate potential subacute hepatotoxic effects of SBH in Sprague-Dawley (SD) rats. Liver sections were examined under light microscopy (40x magnification) to detect cellular or structural abnormalities.

- **Control Group:** Exhibited normal hepatic structure, including radiating hepatocyte cords, intact central veins, and open sinusoids. Hepatocytes appeared polygonal with centrally located nuclei and granular eosinophilic cytoplasm.
- **SBH 500 mg/kg:** Histology was similar to the control group, with preserved hepatic architecture and no evidence of inflammation or degeneration.
- **SBH 750 mg/kg:** Hepatocytes maintained typical morphology, with distinct nuclei and clear cytoplasmic features; sinusoidal spaces remained unobstructed.
- **SBH 1000 mg/kg:** No histopathological abnormalities were detected; hepatic structure remained intact with no signs of fibrosis, necrosis, or inflammatory cell infiltration.

These results confirm that subacute administration of SBH at all tested doses does not induce hepatotoxicity in SD rats. The preserved liver architecture across treatment groups supports SBH's safety for potential therapeutic applications.

### 3.3. Impact of SBH on locomotor activity in an AD rat model

**Figure 3.**
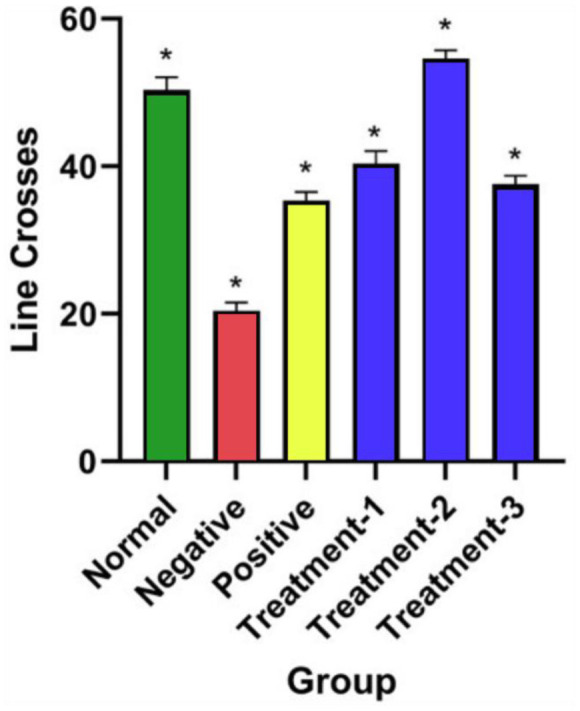
Open Field Test results showing the effect of co-administration of different doses of SBH on rat locomotion activities, represented as the number of line crosses within a period of 10 minutes. **Normal group**: Received saline. **Negative group**: AlCl₃ (150 mg/kg) + D-gal (300 mg/kg). **Positive group**: AlCl₃ (150 mg/kg) + D-gal (300 mg/kg) + DPZ (1.5 mg/kg). **Treatment groups**: **Treatment-1:** AlCl₃ (150 mg/kg) + D-gal (300 mg/kg) + SBH (500 mg/kg); **Treatment-2**: AlCl₃ (150 mg/kg) + D-gal (300 mg/kg) + SBH (750 mg/kg); and **Treatment-3**: AlCl₃ (150 mg/kg) + D-gal (300 mg/kg) + SBH (1000 mg/kg). Data represent mean ± SEM (*n* = 8). The * is the significant *p* < 0.05.

Locomotor activity was assessed using the Open Field Test (OFT) to ensure that co-administration of multiflora SBH did not alter basic motor function or confound behavioural outcomes in the AD rat model. As shown in Figure 3, there were no statistically significant differences in movement across all groups-normal control, negative control (AlCl₃ + D-gal), positive control (donepezil), and SBH-treated groups (500, 750, 1000 mg/kg) with one-way ANOVA confirming *p* > 0.05.

Exploratory parameters, such as line crossings and movement frequency, along with indirect anxiety markers like grooming and faecal output, showed non-significant trends of increased activity in SBH-treated groups, particularly at 750 mg/kg. However, the differences did not reach statistical significance (*p* > 0.05), indicating only mild behavioural modulation. These findings confirm that SBH does not induce sedative or hyperactive effects at the tested doses, validating the integrity of subsequent cognitive assessments. Nonetheless, subtle effects may require more sensitive behavioural assays, such as the Morris Water Maze, for clearer interpretation.

### 3.4. Evaluation of spatial learning and memory following SBH treatment using the morris water maze test

To evaluate the cognitive effects of multiflora SBH in an Alzheimer's-like rat model, spatial learning and memory were assessed using the MWM test a standard hippocampal dependent task for rodent cognitive studies [27],[28]. Rats treated with SBH at 500, 750, and 1000 mg/kg following AlCl₃ and D-galactose induction underwent four days of training to locate a hidden platform, with escape latency recorded. On the fifth day, a probe trial was conducted to evaluate memory retention based on time spent in the target quadrant.

**Figure 4.**
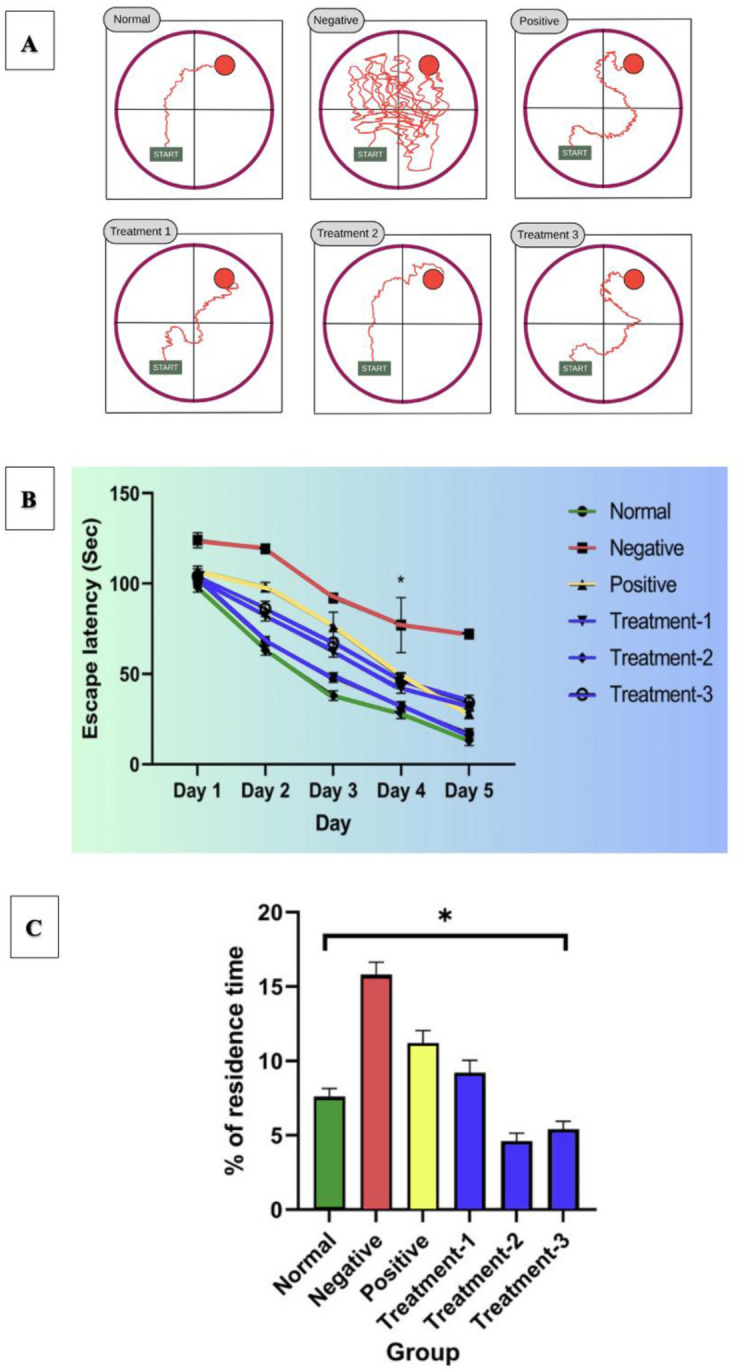
Cognitive performance of rats treated with SBH assessed by the MWM test. **(A)** Representative swim paths recorded on day 4 of the navigation trial for each group. **(B)** Mean escape latency across the 5-day training period. **(C)** Percentage of time spent in the target quadrant during the probe trial. Data are presented as mean ± SEM (*n* = 8). Statistical analysis was conducted using two-way ANOVA with Tukey's post hoc test for (B), and one-way ANOVA with Tukey's post hoc test for (C). * *p* < 0.05 was considered statistically significant.

Figure 4(A) illustrates representative swimming trajectories on the final day of acquisition. Rats treated with 750 mg/kg (Treatment 2) and 1000 mg/kg (Treatment 3) SBH demonstrated more direct navigation paths towards the previous platform location, compared to the negative control group, which showed disoriented swimming, indicating impaired learning. As shown in Figure 4(B), two-way ANOVA followed by Tukey's post hoc test revealed a significant interaction between treatment and training days (*p* < 0.05). Treatment 2 and Treatment 3 groups exhibited significantly reduced escape latencies compared to the negative control and Treatment 1 (500 mg/kg), indicating enhanced spatial learning. No significant difference was observed between Treatments 2 and 3, suggesting a plateau in cognitive improvement at higher doses. In the probe trial as in Figure 4(C), one-way ANOVA showed significant group differences in time spent in the target quadrant (*p* < 0.05). Post hoc analysis confirmed that Treatment 2 and 3 spent significantly more time in the target area than both the negative control and Treatment 1 groups, reflecting improved memory retention.

### 3.5. Hormonal biomarker analysis via ELISA

**Figure 5.**
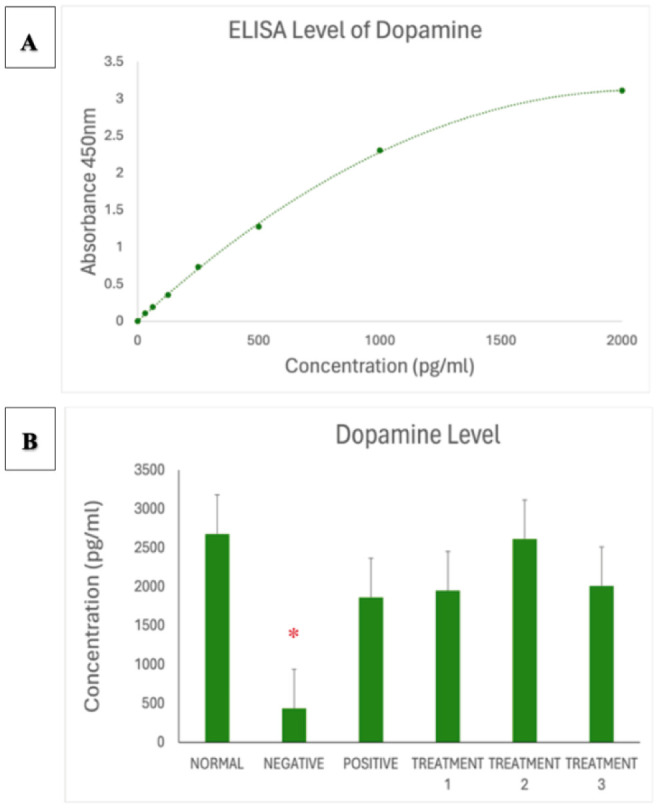
**(A)** Standard curve of Dopamine. Results are expressed as the mean ± SD of standard curves. **(B)** Dopamine ELISA assay showing significantly reduced dopamine concentration in negative SD rats (* *p* < 0.0001), which was ameliorated by treatment with SBH.

As shown in Figure 5(A), the dopamine standard curve validates assay performance. Figure 5B shows that dopamine levels were significantly reduced in the negative control group, consistent with AD-associated neurochemical deficits. SBH treatment restored dopamine levels in a dose-dependent manner, with the 750 mg/kg group showing the most pronounced effect.

**Figure 6.**
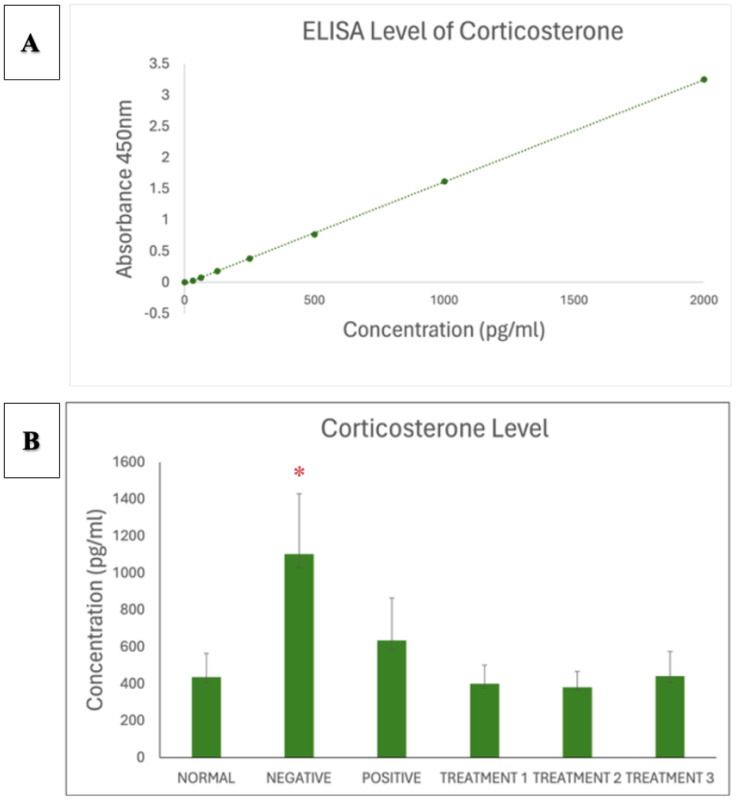
**(A)** Standard curve of corticosterone. **(B)** Corticosterone ELISA assay showing significantly increased corticosterone concentration in negative SD rats (* *p* < 0.0001), which was decreased by treatment with SBH.

Corticosterone standard and serum levels are presented in Figure 6(A) and Figure 6(B), respectively. The negative control group exhibited elevated corticosterone, indicating a heightened stress response. SBH administration significantly reduced corticosterone levels, with the greatest effect observed at 1000 mg/kg.

**Figure 7.**
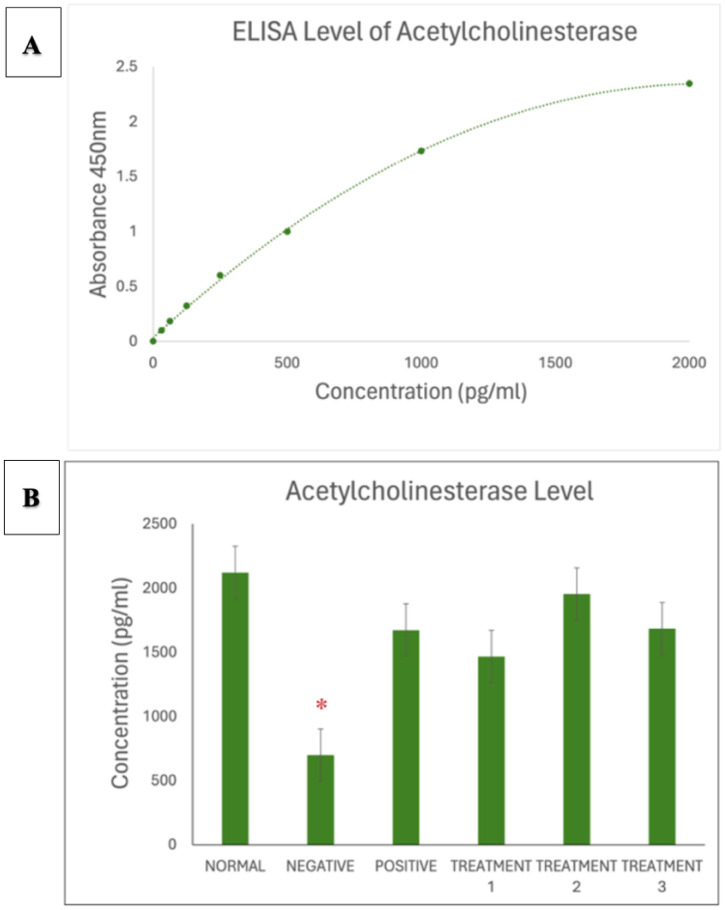
**(A)** Standard curve of Acetylcholinesterase. **(B)** Acetylcholinesterase ELISA assay showing significantly reduced acetylcholinesterase concentration in negative SD rats (* *p* < 0.0001), which was ameliorated by treatment with SBH.

The acetylcholinesterase standard curve is shown in Figure 7(A). As seen in Figure 7(B), AChE levels were markedly decreased in the negative control group. SBH treatment restored AChE activity, particularly at 750 and 1000 mg/kg doses, suggesting improved cholinergic regulation.

**Figure 8.**
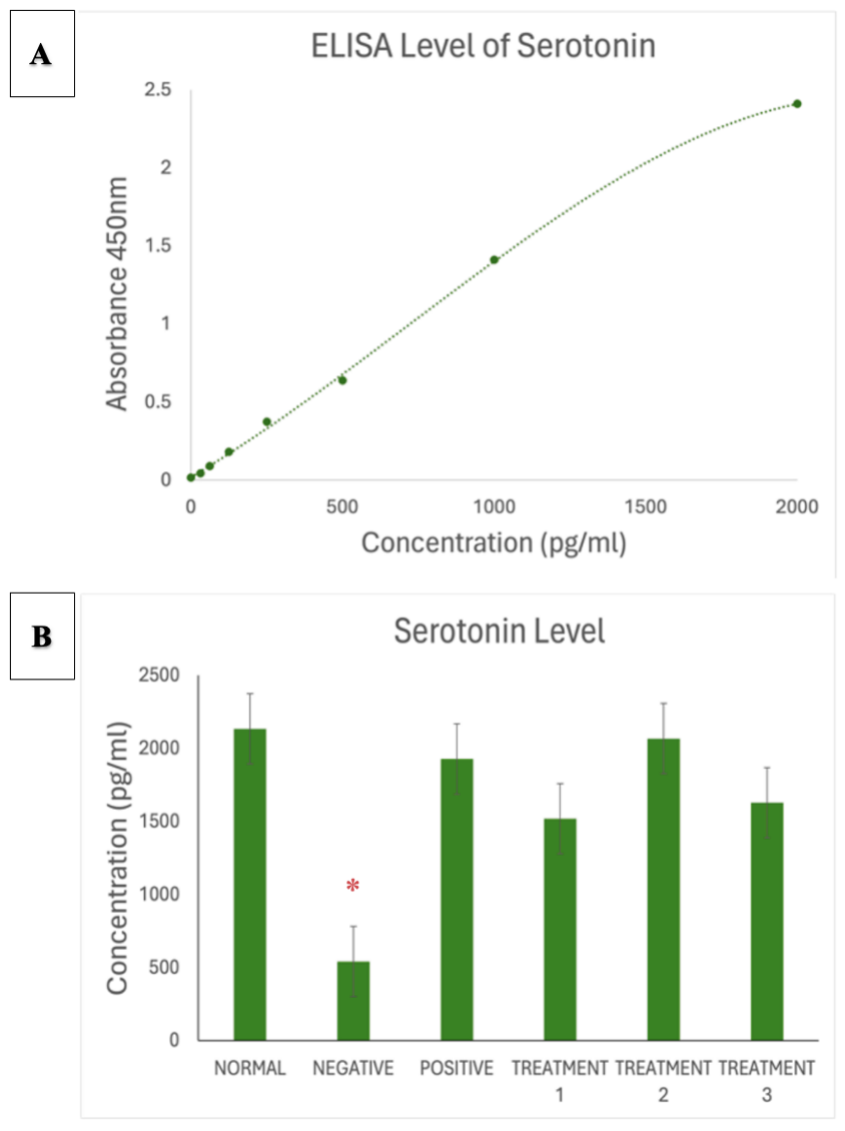
**(A)** Standard curve of Serotonin. **(B)** Serotonin ELISA assay showing significantly reduced serotonin concentration in negative SD rats (* *p* < 0.0001), which was ameliorated by treatment with SBH.

Figure 8(A) displays the standard curve for serotonin. In Figure 8(B), serotonin is significantly lower in the negative control group. SBH-treated rats exhibited dose dependent increases in serotonin levels, with 1000 mg/kg showing the highest restoration, nearing normal control values.

### 3.6. Histopathological analysis of the hippocampus

**Figure 9.**
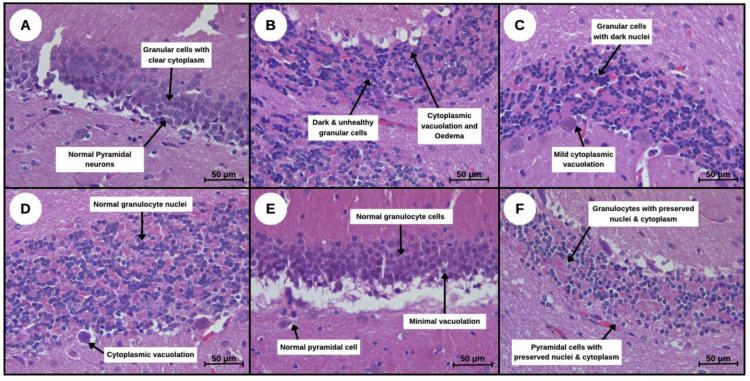
Photomicrographs showing H&E staining of hippocampal sections from all experimental groups (40× magnification). **(A)** Normal group: with intact neurons and clearly visible nuclei. **(B) Negative group**: AD model group shows pyknotic nuclei, irregular cell shapes, cytoplasmic vacuolation, and oedema. **(C) Positive group**: AD + Donepezil (DPZ) group displays mild improvements but notable neurodegeneration. **(D - F) Treatments 1, 2 and 3**: SBH-treated groups (500, 750, and 1000 mg/kg respectively) show improved hippocampal architecture. The SBH 750 mg/kg group **(E)** reveals near-normal neuronal morphology with minimal vacuolation.

Histological examination of H&E-stained hippocampal sections provided further confirmation of Alzheimer-like pathology and the neuroprotective effects of SBH. As shown in Figure 9, the normal control group (A) displays well-preserved hippocampal architecture with clearly defined neurons, visible nuclei, and organised cytoarchitecture. In contrast, the AD model group (B) exhibits marked neuronal damage, characterised by darkly stained, pyknotic nuclei (long arrows), irregular cell morphology, cytoplasmic vacuolation, and signs of pericellular oedema. The donepezil-treated group (C) also shows some degree of structural disruption, although slightly less severe than the untreated AD group. Remarkably, treatment with SBH at doses of 500, 750, and 1000 mg/kg (D, E, and F) results in dose-responsive improvements in cellular morphology. Notably, the 750 mg/kg SBH group (E) exhibits almost normal neuronal structures with reduced vacuolation and minimal degenerative changes. Overall, the histological findings support the biochemical and behavioural data, indicating that SBH mitigates hippocampal neurodegeneration in the AD model.

## 4. Discussion

AD is a multifactorial neurodegenerative disorder marked by progressive cognitive decline, behavioural disturbances, and neuropsychiatric symptoms. Key pathological features include cholinergic dysfunction, oxidative stress, neuroinflammation, and neurotransmitter imbalance [32]–[34]. While acetylcholinesterase inhibitors offer symptomatic relief, they do not halt disease progression and may present safety concerns with prolonged use [35].

Consequently, natural products with antioxidant and neuroprotective properties are being explored as safer alternatives or adjunctive therapies. SBH, rich in flavonoids, phenolic acids, and other bioactive compounds, has shown potential to modulate oxidative damage and neurotransmitter function. This study investigated the therapeutic and safety profile of SBH in an AlCl₃ and D-galactose-induced AD rat model, focusing on subacute toxicity, behaviour, and neurochemical modulation [32].

A critical consideration in evaluating any potential therapeutic agent is its safety, particularly regarding long-term organ health. In this study, subacute toxicity was assessed histologically in kidney and liver tissues following 28 days of oral SBH administration at doses of 500 mg/kg, 750 mg/kg, and 1000 mg/kg. As shown in Figures 1 and 2, histopathological analyses revealed no signs of tissue damage, inflammation, necrosis, or cellular degeneration in either organ across all treated groups. In the kidney, Figure 1, glomerular and tubular structures remained intact in all SBH-treated rats. No interstitial changes or pathological disruptions were observed, indicating that SBH does not pose nephrotoxic risks even at high dosages. This observation aligns with Zulkifli et al., 2023 and Shajahan et al., 2025. [8],[11], who reported that honey's antioxidant content contributes to renal protection by minimizing lipid peroxidation and stabilizing cellular membranes. Similarly, Figure 2 shows liver sections from all treatment groups, displaying preserved hepatic architecture, including polygonal hepatocytes, with centrally located nuclei and intact sinusoids. The absence of hepatocellular swelling, necrosis, or fibrosis is consistent with other studies showing the hepatoprotective effects of honey [36],[37].

Given the liver's role in detoxification and metabolic regulation, these findings support SBH's compatibility with long-term therapeutic use, particularly in patients who may already be on multiple medications. The toxicological profile observed in this study highlights the potential of SBH as a safe candidate for chronic administration, without imposing hepatic or renal stress an essential consideration for any agent aimed at managing neurodegenerative conditions. Behavioural assessments provided insights into the functional outcomes of SBH treatment on locomotion, learning, and memory. Two validated behavioural paradigms were employed: the Open Field Test (OFT) for general locomotor and exploratory behaviour, and the MWM for spatial learning and memory performance. The Open Field Test (OFT) was used to determine whether SBH affects locomotor activity, ensuring that any cognitive effects observed were not confounded by changes in general motor function. Locomotor activity, measured by line crossings, is a standard indicator of exploratory behaviour, anxiety, and CNS stimulation or suppression [26].

As illustrated in Figure 3, there is no statistically significant differences in locomotor activity across all groups, confirming that SBH, at doses of 500, 750, and 1000 mg/kg, does not induce motor impairment or sedation. These findings affirm SBH's CNS safety and validate its use in cognitive testing without behavioural distortion. This aligns with previous studies showing that natural antioxidants like honey enhance brain function without affecting motor behaviour [11],[38]. The negative control group (AlCl₃ + D-gal) displayed reduced line crossings, consistent with AD-related neurotoxicity, oxidative stress, and reduced exploratory behaviour, as reported by Luo and Bustamante-Barrientos [39],[40]. In contrast, donepezil (DPZ), used as the positive control, significantly restored activity, reflecting its known cholinergic enhancement and neuroprotective effect [13],[41].

Among SBH-treated groups, the 750 mg/kg dose showed the most notable improvement in exploratory behaviour, suggesting this dosage may be optimal for supporting CNS function. These results align with Zulkifli et al., 2023 [8], who demonstrated that honey's antioxidant properties can enhance neural stability and reduce behavioural deficits without sedative effects. While the 1000 mg/kg dose also preserved locomotor function, it showed slightly reduced activity compared to 750 mg/kg, suggesting a potential plateau effect, a phenomenon observed in plant-based treatments such as ginseng and curcumin [42]. This supports the idea that moderate doses of SBH may offer maximal behavioural benefits. In summary, OFT findings indicate that SBH does not impair locomotion and may enhance exploratory behaviour at optimal doses. These results support previous evidence on honey's neuroprotective properties and reinforce SBH's potential as a non-sedative therapeutic agent in AD treatment [39].

The MWM was employed to evaluate spatial learning and memory in an AD rat model treated with SBH. The test outcomes, including swimming trajectories, escape latency, and probe trials, demonstrated dose-dependent cognitive improvements, with 750 mg/kg SBH showing the most pronounced effects. These results support SBH's potential as a cognitive enhancer in neurodegenerative conditions. In the acquisition phase, as Figure 4(A) shows, the normal control group displayed direct, goal-oriented swimming paths, while the negative control group (AlCl₃ + D-gal) exhibited disoriented, thigmotaxic behaviour, indicating spatial memory impairment. These patterns reflect typical AD-related cognitive deficits, which is consistent with Shajahan and Morris's [11],[29] findings, noting erratic navigation in AD rodent models. Donepezil-treated rats (positive control) exhibited improved trajectories, reaffirming its efficacy in reversing cognitive deficits via cholinergic enhancement [13]. SBH-treated groups showed progressive improvements, with the 750 mg/kg dose producing the most organised trajectories, suggesting optimal spatial learning restoration. This aligns with findings on other antioxidant-rich natural compounds, where moderate doses yield maximal cognitive benefit [15],[25].

As shown in Figure 4(B), the escape latency analysis further confirmed the dose-dependent cognitive effects. The AD-induced group had significantly prolonged latencies, while the 750 mg/kg SBH group achieved latency times comparable to the positive control. This mirrors earlier research indicating that medium-range doses of neuroprotective agents enhance memory acquisition through improved synaptic plasticity and oxidative stress modulation [15],[35]. In the probe trial, as illustrated in Figure 4(C), rats treated with 750 mg/kg SBH spent significantly more time in the target quadrant, closely approaching the performance of the Donepezil group. This reflects superior memory retention and aligns with prior studies showing that natural antioxidants support hippocampal function and memory consolidation by attenuating neuroinflammation and neuronal loss [11],[15]. Importantly, the 1000 mg/kg dose showed slightly reduced performance compared to 750 mg/kg, suggesting a plateau or mild attenuation of benefit at higher concentrations. This echoes findings from studies on phytochemicals like curcumin and ginseng, where excessive dosages may result in diminished efficacy due to receptor desensitization or antioxidant saturation [42]. In summary, the MWM results show that SBH improves cognitive performance in AD rats in a dose-dependent manner, with 750 mg/kg emerging as the most effective dose. These findings strongly align with previous studies on neuroprotective natural products and support SBH's role as a promising, non-toxic therapeutic candidate for managing cognitive decline in AD.

Importantly, the improvements observed in MWM performance were not confounded by changes in locomotor ability. During the OFT and MWM trials, no abnormal motor behaviour, hyperactivity, or impairment was observed across experimental groups. The increased line crossings and centre entries in SBH-treated rats remained within physiological norms, indicating enhanced exploratory motivation rather than excessive locomotion. Moreover, swim speeds were comparable among all groups, confirming that the superior MWM performance in SBH-treated rats particularly at 750 mg/kg reflects genuine cognitive enhancement rather than altered motor activity. This distinction reinforces the conclusion that SBH's effects are primarily cognitive, potentially mediated through neurotransmitter modulation and antioxidant mechanisms.

The ELISA results offer insight into the neurochemical modulation exerted by SBH in the AD rat model. Key biomarkers assessed dopamine, corticosterone, and acetylcholinesterase (AChE), and serotonin reflected neurotransmission, stress response, and cognitive regulation. SBH exhibited dose-dependent therapeutic effects across these biomarkers, with the 750 mg/kg dose yielding the most favourable outcomes. As shown in Figure 5(B), dopamine levels were significantly reduced in the AD model group, aligning with other findings that neurodegeneration disrupts dopaminergic transmission, contributing to memory and motor deficits [43]. SBH treatment restored dopamine levels in a dose-dependent manner, particularly at 750 mg/kg, which is consistent with prior reports that antioxidant-rich compounds can protect dopaminergic neurons and support cognitive recovery [15]. These findings indicate that SBH may help alleviate AD-related cognitive symptoms by restoring dopaminergic function. Similarly, Figure 6(B) shows that corticosterone, an indicator of neuroendocrine stress, was significantly elevated in the negative control group, mirroring the chronic stress response seen in AD pathology [44],[45]. SBH treatment reduced corticosterone levels across all doses, with the 750 mg/kg group showing a near-normal profile. This suggests that SBH may exert anxiolytic and anti-stress effects, a result that parallels studies demonstrating the stress-reducing capabilities of natural antioxidants in neurodegenerative models [8],[45].

For AChE in Figure 7(B), the AD model group showed reduced levels, reflecting disrupted cholinergic function, a hallmark of AD progression [46]. SBH significantly increased AChE levels, particularly at 750 mg/kg, supporting the restoration of cholinergic signalling. This aligns with studies by Shaikh and Walczak-Nowicka & Herbet [15],[42], which showed improved cholinergic function and memory performance following antioxidant intervention. The enhancement of AChE activity further validates SBH's role in improving synaptic efficiency and cognitive outcomes. As illustrated in Figure 8(B), serotonin, which is crucial for mood and cognition, was markedly decreased in the AD group, which is consistent with serotonergic deficits commonly observed in AD patients [47]. SBH treatment, especially at 750 mg/kg, significantly elevated serotonin levels, suggesting its potential to alleviate mood disturbances and cognitive dysfunction. These effects mirror findings from Zulkifli and Trillo [8],[48], who highlighted the antidepressant-like and neuroprotective properties of natural flavonoids. In summary, SBH demonstrated beneficial modulation of key neurochemical markers associated with AD. By increasing dopamine, serotonin, and AChE, while reducing corticosterone, SBH reinforces its potential as a multi-targeted, non-toxic therapeutic agent [11],[49],[50]. These biochemical findings support behavioural improvements observed in the OFT and MWM tests, and align with the literature on the neurotherapeutic effects of natural compounds in AD models.

As shown in Figure 9, histological findings in this study provided anatomical validation of the AD model and supported the observed behavioural and biochemical changes. In untreated AD-induced rats, hippocampal sections revealed densely stained neurons, pericellular vacuolation, and disrupted cytoarchitecture hallmarks of oxidative stress-induced neurodegeneration. These structural alterations are consistent with pathological features reported in similar chemically induced AD models, where neuronal shrinkage and loss of hippocampal integrity were observed [27].

In contrast, SBH-treated groups, as shown in Figure 9, exhibited preserved neuronal structures, with the 750 mg/kg group showing the most pronounced neuroprotective effect. Neurons in this group appeared structurally intact, with reduced vacuolation and minimal cytopathological features, suggesting that SBH attenuates histological damage. These improvements may be attributed to the antioxidant and anti-inflammatory properties of SBH's bioactive compounds, which help stabilise neuronal membranes and reduce oxidative burden [4],[51]. Interestingly, while donepezil-treated rats showed modest behavioural improvement, histological analysis revealed ongoing neuronal degeneration, supporting findings that donepezil may improve symptoms without halting underlying structural damage [52]. Overall, the histological outcomes reinforce the behavioural and biochemical results, indicating that SBH, particularly at 750 mg/kg, offers significant structural protection against AD-related hippocampal damage. The dose-responsive differences observed among the SBH-treated groups suggest an optimal therapeutic window at 750 mg/kg.

A notable finding of this study is the non-linear, biphasic dose-response pattern, in which the 750 mg/kg SBH dose consistently produced the most significant improvements across behavioural performance, neurotransmitter levels, and hippocampal integrity. Such biphasic responses are well-documented in natural products rich in polyphenols and bioactive compounds, where moderate doses often yield superior therapeutic effects compared to higher concentrations [53],[54]. This phenomenon may be attributed to several mechanisms, including receptor desensitization, metabolic saturation, competition among bioactive constituents, or reduced bioavailability at higher doses due to compound interactions. The slightly reduced efficacy observed at 1000 mg/kg SBH in this study aligns with these possibilities. Therefore, in future investigations, researchers should explore a broader dosing range, including lower (250 mg/kg) and higher doses (1250–1500 mg/kg) to better define the optimal therapeutic window and further elucidate the mechanistic basis of this biphasic effect. Such dose-optimization studies will be crucial for translating SBH into clinically relevant formulations.

Chemical characterization of the SBH used in this study represents an important limitation worth acknowledging. Although SBH is widely reported to contain abundant flavonoids, phenolic acids, organic acids, and distinctive sugars with neuroactive and antioxidant properties [38],[55], we did not perform direct phytochemical profiling of the *Heterotrigona itama* honey sample used. Moreover, researchers using HPLC and LC-MS have demonstrated that this species' honey contains gallic acid, caffeic acid, quercetin, and other phenolic constituents linked to neuroprotection, anti-inflammatory effects, and cholinergic modulation [55]–[57]. However, without chemical analysis of the specific batch used in this experiment, the contribution of individual bioactive compounds to the observed therapeutic effects remains speculative. In future studies, researchers should incorporate comprehensive chemical profiling, including total phenolic content, flavonoid quantification, and LC-MS fingerprinting, to directly correlate SBH's phytochemical composition with its behavioural, neurochemical, and histological outcomes. Such data would significantly strengthen mechanistic interpretation and support the standardization of SBH for potential nutraceutical development.

While the neuroprotective effects of SBH observed in this study appear promising, alternative explanations must be considered. Improvements in behavioural performance and neurochemical balance may not be solely attributable to specific bioactive compounds; rather, they could also reflect non-specific antioxidant or metabolic support effects, or even reduced stress responses following daily oral administration. Additionally, variations in gut-brain axis modulation, influenced by SBH's natural sugars and prebiotic components, may have indirectly affected central nervous system function [58].

In addition to the therapeutic findings, we incorporated a subacute toxicity evaluation in accordance with OECD Test Guideline 425 (2022) to ensure the short-term safety of SBH at the administered doses. No adverse behavioural signs, mortality, or histopathological abnormalities were detected in the liver and kidney tissues of rats receiving 500, 750, and 1000 mg/kg SBH, supporting its classification as a non-toxic natural product under subacute conditions. However, we recognise that long-term administration is clinically more relevant for neurodegenerative diseases, where treatment often extends for months or years. The lack of chronic toxicity assessment therefore represents a limitation of this study. In future work, researchers should include extended exposure protocols to evaluate body and organ weight progression, hematological and biochemical parameters, and chronic liver and kidney histopathology. Such studies will be fundamental to establishing a comprehensive safety profile for SBH and determining its suitability for long-term therapeutic use in AD.

Despite these possibilities, the consistency of outcomes across behavioural, biochemical, and histological domains suggests a true therapeutic effect. The findings highlight SBH's potential as a safe, natural intervention for early-stage AD or mild cognitive impairment. However, translating these results to clinical settings requires caution. Factors such as dosage optimization, long-term safety, and compound standardization must be thoroughly investigated. Human trials examining cognitive outcomes and biomarker changes after SBH supplementation would be essential for validating its applicability in clinical practice. The observed neurotherapeutic effects of SBH in this study aligns with its longstanding use in traditional medicine across Southeast Asia. In Malay and Indonesian ethnomedicine, SBH is frequently utilised as a natural remedy to boost vitality, alleviate fatigue, and support cognitive function in the elderly [8],[59]

These practices, passed down through generations, suggest a cultural recognition of SBH's health benefits, particularly in the context of aging and memory. Our findings provide scientific validation for these traditional claims, highlighting the potential of SBH as a natural intervention for neurodegenerative conditions like AD. This reinforces the importance of integrating ethnopharmacological knowledge into biomedical research, especially when exploring multifunctional natural products with both historical and therapeutic relevance. While we found promising behavioural, biochemical, and histological outcomes, we did not include direct molecular assessments of Alzheimer's hallmark pathologies, such as amyloid-beta accumulation or tau hyperphosphorylation. These analyses, while highly valuable, fall beyond the scope and major objectives of this preclinical design, where we focused on establishing therapeutic efficacy and safety. Nevertheless, we recognise this as a key direction for future work and plan to incorporate molecular and immunohistochemical evaluations of Aβ and phosphorylated tau expression in subsequent studies to further elucidate the mechanisms underlying SBH's neuroprotective potential.

Furthermore, we acknowledge that the AlCl₃ + D-galactose model mainly reflects features of sporadic AD, such as oxidative stress, neuroinflammation, and cholinergic dysfunction, as widely reported in chemical induction models [17],[60], but does not fully replicate the progressive amyloid and tau pathology seen in human patients. Therefore, in future studies, we will aim to validate SBH's therapeutic efficacy in a transgenic Alzheimer's model, such as the APP/PS1 mouse line, which is characterised by genetically driven Aβ plaque deposition and progressive neuropathology [61],[62], which more closely mirrors the heritable and progressive nature of AD pathology. Evaluation in such models will provide a more comprehensive translational assessment and further confirm SBH's potential in complex neurodegenerative settings.

In future studies, researchers should explore molecular mechanisms in greater detail, such as oxidative stress markers, neuroinflammatory signalling, and mitochondrial function. Furthermore, integrating SBH into combinatory therapies with drugs like donepezil may provide synergistic benefits while minimizing side effects. Collectively, this study lays the groundwork for advancing SBH as a candidate for nutraceutical development in neurodegenerative disease management.

## 5. Conclusions

This study highlights the therapeutic potential of SBH in attenuating AD-like pathology. SBH administration notably improved cognitive function, behavioural performance, neurochemical homeostasis, and histological analysis in a rat model of AD, indicating its ability to counteract the cognitive and psychological deficits associated with neurodegeneration. Owing to its rich profile of neuroactive and antioxidant compounds, SBH emerges as a promising natural candidate for adjunctive therapy in the management of AD. These findings provide a compelling foundation for future research to elucidate its precise mechanisms of action and evaluate its translational potential in clinical settings.

## Use of AI tools declaration

The authors declare that Artificial Intelligence (AI) tools were used solely for language editing, grammar correction, and improvement of clarity and readability of the manuscript. The scientific content, data interpretation, analyses, and conclusions were entirely developed by the authors, who take full responsibility for the integrity and originality of the work.
